# Butylbenzene and *tert*-Butylbenzene—Sorption on Sand Particles and Biodegradation in the Presence of Plant Natural Surfactants

**DOI:** 10.3390/toxins10090338

**Published:** 2018-08-22

**Authors:** Agata Zdarta, Amanda Pacholak, Marta Galikowska, Wojciech Smułek, Ewa Kaczorek

**Affiliations:** Institute of Chemical Technology and Engineering, Poznan University of Technology, Berdychowo 4, 60-965 Poznan, Poland; amanda.d.pacholak@doctorate.put.poznan.pl (A.P.); marta.galikowska@gmail.com (M.G.); wojciech.smulek@put.poznan.pl (W.S.); ewa.kaczorek@put.poznan.pl (E.K.)

**Keywords:** surfactants, butylbenzene, *tert*-butylbenzene, biodegradation, sand

## Abstract

The effects of hydrocarbons sorption on sand and saponins presence in the system on butylbenzene and *tert*-butylbenzene biological degradation was investigated. Additionally, the impact of saponins-containing plant extracts on environmental microorganisms was studied. Results of cell surface property measurements in samples with saponins only revealed changes in cell surface hydrophobicity, electrokinetic potential and membrane permeability when compared to corresponding values for glucose-grown microbes. Subsequently, in sorption experiments, the hydrocarbon adsorption kinetics in bacteria-free samples were better explained with the pseudo-second order kinetic model as compared to the pseudo-first order and intraparticular diffusion models. Moreover, the equilibrium data fitted better to the Freundlich isotherm for both benzene derivatives. In the samples combining hydrocarbons sorption and biological degradation in the presence of saponins, alkane-substituted hydrocarbons removal was accelerated from 40% to 90% after 14 days and the best surfactant in this aspect was *S. officinalis* extract.

## 1. Introduction

Despite growing awareness of societal and legal regulations and the use of innovative techniques in industry and transport, environmental contamination with toxic chemical compounds still occurs [1,2]. A number of remediation techniques have been developed for the treatment of contaminated sites, including physicochemical and biological methods [3,4,5,6]. The latter have attracted considerable attention, due to relatively low costs and negligible interference with the ecosystem [7].

However, the complexity of processes involved in biological degradation of xenobiotics creates the issue for deep investigation focusing on various aspects of that problem [8,9]. The microbiological potential of the contaminated ecosystem [10], accessibility of additional nutrients and oxygen [11], and bioavailability of pollutants [12] are the examples of factors which have strong impacts on biodegradation efficiency.

Among various parameters which influence the biodegradation of hydrocarbons in soil, the problems of sorption and desorption of a chemical compound on solid particles of a matrix cannot be ignored. Strong binding of hydrocarbon molecules to the soil changes its properties remarkably and strongly affects the activity of autochthonic micro- and macro-organisms [13]. Hydrophobic compounds, such as aromatic hydrocarbons, are very poorly water soluble and have a strong affinity to soil particles [3,14]. As a result, their bioavailability to microorganisms is limited. Hence, the phenomenon of diffusion, which is strongly inhibited by substantial sorption, is often the rate-determining step of the bioremediation process [15,16]. Butylbenzene and *tert*-butylbenzene’s production and uses as chemical intermediates during the synthesis of chemicals and raw materials for liquid crystal, and use as a solvent, may result in its release to the environment through various waste streams. They might adsorb to soil particles and suspended solids and sediment in water based upon estimated high soil distribution coefficient (K_OC_), thus reducing their bioavailability.

An effective remedy for significant binding of hydrophobic pollutants to soil particles can be the use of surfactants. Introducing them to the contaminated environment can bring a number of beneficial effects [9]. The presence of surface active agents facilitates the desorption of (adsorbed) soil contaminants and their migration to microbial cells [17,18]. Furthermore, the surfactants themselves influence the bacterial cells, leading to the reconstruction of their surface and change in bioavailability of the biodegraded compound [19,20].

On the other hand, it should be highlighted that the surfactants introduced into the environment may become an additional threat not only to microorganisms, but also to higher organisms. Therefore, it is desirable to use, in bioremediation techniques, surfactants which are as non-toxic as possible and undergo biodegradation without generation of toxic intermediates [9].

These requirements are met by natural surfactants, among which biosurfactants, produced mainly by microorganisms, enjoy increasing attention. They are able to reduce surface tension and allow generation of stable multiphase systems [21]. A number of studies have shown that biosurfactants, e.g., rhamnolipids, show properties comparable to those observed for synthetic surfactants [22,23]. The disadvantage of biosurfactants, however, is the cost of their production, which is related to harvesting large scale microbiological cultures [24]. An alternative to biosurfactants may be surfactants of plant origin [25,26]. They include saponins, which effectiveness as a factor improving the biodegradation of persistent contaminants has been confirmed by several studies in bioremediation of diesel oil and polycyclic aromatic hydrocarbons (phenanthrene and pyrene) [27,28,29,30,31]. In the mentioned studies, surfactant addition enabled to removal of around 34% of phenanthrene and over 50% of diesel oil and pyrene after 7 days, and 88% of diesel oil after 14 days.

Compared to other groups of surfactants, the application of saponins in biodegradation processes is relatively scarcely studied, which raises the requirement of intensive research in this area. The interaction of plant-derived surfactants has not been extensively studied in relation to biosurfactants–saponins competitiveness, so the influence of an additional bioactive compound on cell surface properties and biodegradation ability was not examined. Also, the mechanism of the cell–sand–surfactant–hydrocarbon interaction remains undescribed. Whilst some research has been carried out on the degradation of benzene, there have been a few empirical studies of biological decomposition and sorption of alkane-substituted benzene derivatives. Hence, the aim of the study was to describe the kinetics and the role of butylbenzene and *tert*-butylbenzene sorption onto sand particles as well as biodegradation of these hydrocarbons. In parallel studies, cell surface properties of microbial strains capable of degrading such compounds were performed. In addition, the influence of plant extracts containing saponins on the above-mentioned processes was investigated.

## 2. Results and Discussion

### 2.1. Microbes Surface Properties

**Figure 1:**
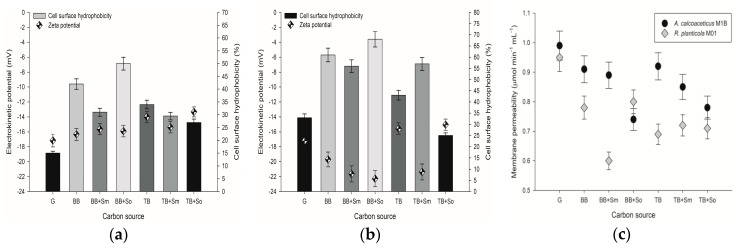
Results of cell surface hydrophobicity and electrokinetic potential measurements of: (**a**) *A. calcoaceticus* M1B; (**b**) *R. planticola* M01; cultured on different carbon sources; (**c**) membrane permeability of analyzed strains cultured on different carbon sources. Letters on the x-axis states for different carbon sources in the culture: G–glucose, BB–butylbenzene, TB–*tert*-butylbenzene, Sm–*Sapindus mukorossi* extract, So–*Saponaria officinalis* extract. Each test was performed in triplicate.

**Figure 2:**
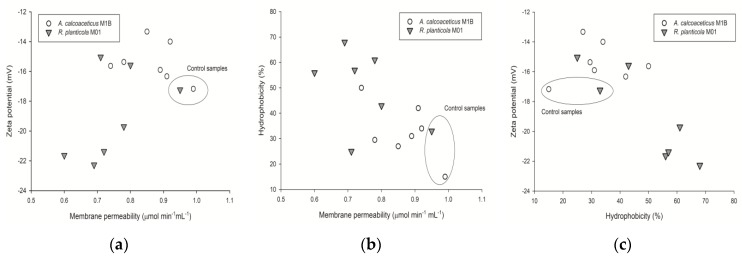
Plots of zeta potential (**a**) and hydrophobicity (**b**) as a function of membrane permeability; (**c**) plot of zeta potential and cell surface hydrophobicity; measured for cells cultured on different carbon sources. Glucose samples are marked as a control sample in the graphs.

Cell surface properties that may influence the interaction of bacterial cells with solid matrices and hydrocarbons are considered to be vital in environmental bioremediation [32]. In this study three various cell surface properties: cell surface hydrophobicity, cell membrane permeability, and zeta potential of microbial suspension, were measured after contact of two selected microbial strains with butylbenzene (BB) and *tert*-butylbenzene (TB). Moreover, the influence of the addition of two plant extracts containing saponins on the properties in question was examined. The results are presented in Figure 1 and Figure 2.

For *Acinetobacter calcoaceticus* M1B (Figure 1a) an increase in cell surface hydrophobicity (CSH) was noticed in each sample containing hydrocarbons in comparison with that of the cells grown on glucose. This strain is capable producing bioactive compounds, as proved in our previous experiments (hemolysis test on blood agar plates, data not shown). The highest value of hydrophobicity (50%) was observed in the cells grown on butylbenzene with the addition of *Saponaria officinalis* extract. This result was 8% higher than the one observed in the samples without the addition of surfactants. Microbial cells grown with the addition of butylbenzene and *Sapindus mukorossi* extract were characterized by significantly lower hydrophobicity. However, different results were obtained for the cells which were grown on *tert*-butylbenzene; the addition of each plant extract resulted in a slight decrease in their hydrophobicity. This indicates that the addition of surfactants to a microbial system does not always modify the hydrophobicity of microbial cells in the same manner. The mechanism is equivocal. On one hand, the surfactant molecule may embed in the cellular surface which results in increased or decreased cell surface hydrophobicity. On the other hand, the presence of surfactant molecules might regulate other cell surface properties, depending on the structure of the cell [33]. Also, the competition mechanism between self-produced and plant-derived surfactants might be important in this case, resulting in various cells reactions on saponins and hydrocarbons in the tested system. The important aspect which could also influence cell surface hydrophobicity may be the source of carbon and energy. In our experiments the hydrocarbons with a branched side chain could have prevented substantial hydrophobicity changes [34]. It has been found by Pei et al. [35] that natural surfactants contribute to increases in the hydrophobicity of cell surfaces and therefore, improve bioavailability of the contaminant to be degraded. However, in our study the presence of plant extracts containing surfactants did not always result in the increased cell surface hydrophobicity. What is more, for *A. calcoaceticus* M1B the saponins from *S. mukorossi* caused a decrease in microbial hydrophobic properties. These results share familiarity with those from the studies of Kaczorek et al. [36] who found out that soap nuts extract reduced hydrophobicity of *Raoultella planticola* WS2 and Zhao et al. [37], who found out that rhamnolipids, which are also natural sugar based surfactants but of bacterial origin, reduced the hydrophobicity of the initially hydrophobic *B. subtilis* BUM strain. In other samples from our experiments such a modification has been also observed. This lack of correlation can be attributed to different properties of both microbial cells and surfactants used.

Focusing on the zeta potential results, no significant changes were observed for *A. calcoaceticus* M1B samples with butylbenzene, being the source of carbon and energy, in comparison with the sample containing the microbial strain grown on glucose (Figure 1a). Slightly higher zeta potential was noted for the samples with *tert*-butylbenzene. On the other hand, the surfactants present in the plant extracts studied did not modify the zeta potential noticeably. In general, the zeta potential (ζ) indicates the microbial suspension stability, based on the electrostatic repulsion between cells. The higher absolute value of ζ, the stronger the electrostatic repulsion that can prevent aggregation by keeping colloidal particles well separated from each other and from surfaces, and the more stable the suspension is. The results presented show that the addition of surfactants did not improve the cell suspension stability and cells might aggregate and adhere to surfaces. This might indicate that in the case of *A. calcoaceticus* M1B the surfactants are more likely to interact with the hydrocarbons particles by their micellization and emulsification, than to adhere to the microbial cells. This also accords with our earlier observations, which showed that saponins addition enhanced cell aggregation [28]. This observation supports results from membrane permeability measurements.

The results of experiments describing cell surface properties obtained for the *R. planticola* M01 strain are shown in Figure 1b. No significant correlation was found between the zeta potential and cell surface hydrophobicity. In general, the hydrophobicity of cell surfaces was appreciably higher when the cells grew on hydrocarbons than on glucose. In the sample with addition of butylbenzene as the sole source of carbon and energy, the hydrophobicity of cell surfaces was 61%. When *S. mukorossi* extract was additionally present in the microbial culture, the value decreased to 56%, however, in the presence of *S. officinalis* extract it increased to 68%. The opposite direction of changes was observed when *tert*-butylbenzene was used as a source of carbon and energy in microbial cultures. In contrast to the results obtained for *A. calcoaceticus* M1B, the values of the zeta potential obtained for *R. planticola* M01 were fluctuating significantly depending on the sample (Figure 1b). However, the most evident changes were observed for the samples containing only butylbenzene with *S. officinalis* as well as *tert*-butylbenzene with *S. mukorossi*.

As for the results of membrane permeability (Figure 1c) obtained for *A. calcoaceticus* M1B, the highest value was observed for microbial cells cultured with the addition of glucose. Cellular membrane permeability is considered to be an important parameter which can regulate the uptake of pollutants to be degraded by microbial cells. Higher cell membrane permeability usually corresponds to better uptake and degradation of hazardous organic compounds. However, it can indicate the rupture of the membrane and following cell damage. Slight decreases for *A. calcoaceticus* M1B might be correlated to cell membrane sealing to reduce the toxic effect of hydrocarbons, as the zeta potential and hydrophobicity values also did not change noticeably. This suggests different mechanism of microbial cells–hydrocarbons/surfactant interaction in the case of this strain, compared to results presented below for *R. planticola* M01. As for the results of inner membrane permeability, in each sample containing hydrocarbons this feature was lower than in the sample with glucose (Figure 1c). Moreover, the presence of plant extracts in microbial cultures contributed to further lowering of the values of membrane permeability of bacterial cells.

In *R. planticola* M01 samples containing only butylbenzene with *S. officinalis* as well as only *tert*-butylbenzene with *S. mukorossi*a decrease in bacterial membrane permeability in the presence of both hydrocarbons was noticed (Figure 1c). The largest decline of permeability was noted for butylbenzene with *S. officinalis* and *tert*-butylbenzene with *S. mukorossi* setups (from 0.98 to 0.74 and 0.85 μmol min^−1^ mL^−1^, respectively). Collating these findings with cell hydrophobicity and zeta potential changes a clear impact of used surfactants and hydrocarbons towards CSH increase and ζ decrease can be noticed. This might be due to the embedding of surfactants in *R. planticola* M01 cells membranes (Figure 2).

Hua et al. [38] have measured the impact of the addition of Tween 60 on the zeta potential of microbial suspensions during the degradation of hexadecane by two *Pseudomonas aeruginosa* strains. For both strains, the zeta potential was higher in the samples with the addition of the surfactant. In our studies, the zeta potential in the systems with surfactants was mostly lower than in the samples without the saponins added. This can be explained by the nature of surfactant used. Tween 60 as synthetic compound substantially changed the properties of microbial suspensions. However, compounds of natural origin used as surfactants in our study did not modify the charge of bacterial cells. Furthermore, studies of Yalҫin et al. [39] have shown that the addition of a surfactant substantially decreased the zeta potential of a microbial suspension and increased the cell surface hydrophobicity. Our results share similarities with those of Yalҫin et al. Although the modifications were not significant, the same directions of changes in both parameters were observed (Figure 2c).

From the plots shown in Figure 2, some correlation between cell membrane permeability and zeta potential as well as hydrophobicity and zeta potential might be found. The results obtained for both strains cultured with hydrocarbons and hydrocarbons and surfactants are concentrated in separated areas and distanced from those obtained for control samples.

### 2.2. Sorption Kinetics

The behavior of the hydrocarbon sorption process was analyzed using the pseudo-first order [40], pseudo-second order [41] and intraparticle diffusion [42] models. Then, the distribution of hydrocarbons between solid phase and solution at a certain temperature when the equilibrium was reached was characterized by the adsorption isotherms. The fit of experimental data of adsorption to the Freundlich [43] and Langmuir [44] models was checked and the goodness of the fit was evaluated. Equations used for the kinetic and isotherms adjustment are given in Table 1.

**Figure 3:**
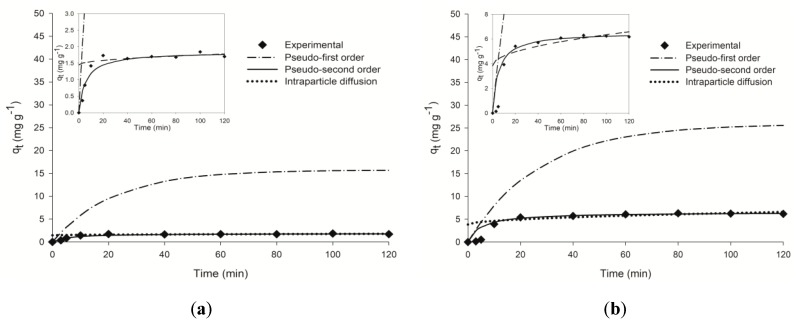
Adsorption rate curves for: (**a**) butylbenzene and (**b**) *tert*-butylbenzene, measured (diamonds) and predicted (lines) from the sorption models.

The kinetics of adsorption were investigated in order to understand the rate and mechanism of adsorption. Figure 3 shows the variation in the amounts of adsorbed hydrocarbon (*q_t_*) as a function of time.

The plot of experimental points as a function of the sets of data calculated from the constants of kinetic models indicates that the adsorption of hydrocarbons was quite fast initially and became slower with time, reaching equilibrium after 80 min for *tert*-butylbenzene and 60 min for butylbenzene. This might be due to higher availability of the uncovered surface area of the adsorbent at the beginning of the process and diminishing number of available active sites with time, which is reflected by the plateau line indicating the equilibrium state [45]. This also accords with the earlier observations [46,47]. In addition, the plots in Figure 3 are the best described by the pseudo-second order model. The kinetic parameters calculated from the pseudo-first order, pseudo-second order and intraparticle diffusion equations for both hydrocarbons are summarized in Table 2.

The *R*^2^ values were 0.924 and 0.972 for butylbenzene and *tert*-butylbenzene, respectively, for pseudo-first order model, and the calculated *q_e_* values did not correspond to the experimental ones, suggesting that adsorption of these hydrocarbons on sand was not a first-order reaction. On the other hand, all the experimental data showed satisfying correlation coefficient values (over 0.99) with the pseudo-second order model. Moreover, the calculated *q_e_* values corresponded well with experimental data, indicating that the sorption process of both benzene derivatives was the pseudo-second order. This suggests the occurrence of chemisorption in addition to physisorption of the sorbate, so the intraparticle diffusion model was tested. However, the obtained correlation coefficient values indicated that intraparticle diffusion model was not suitable to describe the butylbenzene and *tert*-butylbenzene sorption on sand, while *R*^2^ values were very low. Similar observations have been reported by [48] for chlorobenzenes sorption onto cetyltrimethylammonium bromide (CTMAB)-bentonite, while intraparticle diffusion played a significant role for sorption of the same compounds onto the more tight and ordered structure of CTMAB-modified kaolinite. It is also worth mentioning that the pseudo-second order model has been reported as applicable for the sorption of chlorobenzenes onto CTMAB-modified bentonite and kaolinite [48] and BTX sorbed onto purolite-macronet^®^MN-202 spherical resin particles, Claytone-40 clay and activated carbon [49] as well as onto Na-P1zeolite [45] and raw and thermally-modified diatomite [50].

To describe the experimental adsorption isotherms, the Freundlich and Langmuir models were applied expressed by the equations given in Table 1. The Freundlich model describes the relationship between the sorption and density of a compound on a solid phase and their equilibrium concentration in liquid phase. The logarithmic form of the Freundlich equation was employed to establish a mathematical relationship between the adsorbed and free hydrocarbons. The values of *K_F_* and 1/*n* correspond to the intercept and slope of the curves, respectively.

The Langmuir isotherm is based on the assumption that a given surface contains a finite number of adsorption sites of uniform energies of adsorption and at maximum adsorption only a monolayer is formed, with no transmigration of adsorbate into the pores of the adsorbent surface and no migration of the adsorbate between adsorption sites until they are desorbed. The linearized form of the Langmuir isotherm model was used to determine the equilibrium data. The parameters and constants appearing in the Langmuir equation were estimated from linear regression by plotting *C_S_*/*q_e_* vs. *C_S_*. The calculated parameters for both applied adsorption isotherms models are presented in Table 3.

As follows from Table 3, the Freundlich model provides the best fit to the experimental data, which is confirmed by very good correlation coefficient values (0.987 and 0.997 for butylbenzene and *tert*-butylbenzene, respectively). The values of 1/*n* were lower than one for both tested chemicals, suggesting that their adsorption onto the sand particles was favorable, whereas low *K_F_* parameter values indicate a low adsorbent capacity [50]. Moreover, higher values of b constant for *tert*-butylbenzene might be attributed to a higher degree of surface coverage and also correspond to smaller sorption capacity of this compound. Several studies on the sorption of BTX onto different sorbents have reported a better fit of experimental data either to Langmuir or Freundlich isotherm. Benzene, toluene and xylene mixture sorbed onto modified zeolite [45] and purolite-macronet^®^MN-202 spherical resin particles, Claytone-40 clay and activated carbon [49] followed the Langmuir isotherm, while raw and thermally modified diatomite [50] sorption results corresponded better to the Freundlich isotherm. Not only might the adsorption capacity of the sorbent, but also the partitioning mechanism of the adsorbate, affect the results [45]. It should be also noted that the results of benzene, toluene, ethylbenzene, xylene, trichloroethylene (TCE) and perchloroethylene (PCE) sorption onto sandy soils presented by Albergaria et al. [51] demonstrated suitability of the Freundlich model to describe the experimental data.

### 2.3. Hydrocarbons Biodegradation

**Figure 4:**
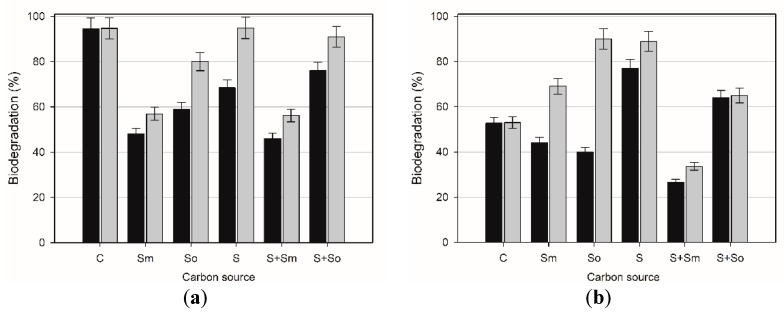
*A. calcoaceticus* M1B biodegradation rates after 7 (black bars) and 14 (grey bars) days of butylbenzene (**a**) and *tert*-butylbenzene (**b**) in different cultures types: C—control sample with hydrocarbons only; Sm—with *S. mukorossi* extract; So—with *S. officinalis* extract; S—with sand only; S+Sm—with sand and *S. mukorossi* extract; S+So—with sand and *S. officinalis* extract. Each test was performed in triplicate.

**Figure 5:**
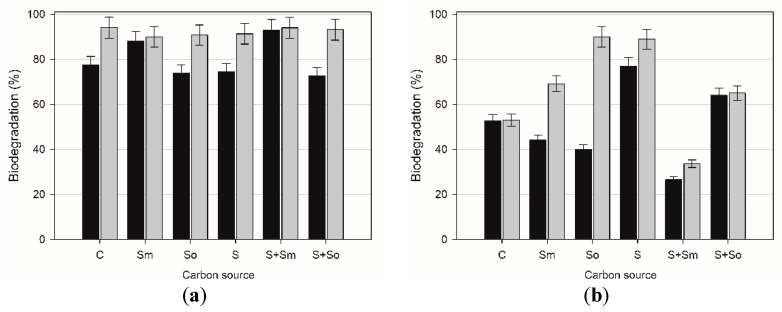
*R. planticola*M01 biodegradation rates after 7 (black bars) and 14 (grey bars) days of exposure to butylbenzene (**a**) and *tert*-butylbenzene (**b**) in different culture types: C—control sample with hydrocarbons only; Sm—with *S. mukorossi* extract; So—with *S. officinalis* extract; S—with sand only; S+Sm—with sand and *S. mukorossi* extract; S+So—with sand and *S. officinalis*extract.

This paper examines the influence of solid matrix and plant extract containing saponins on biodegradation of butylbenzene and *tert*-butylbenzene. The experiments were carried out with the use of *R. planticola* M01 and *A. calcoaceticus* M1B strains. The tests results are shown in Figure 4 and Figure 5.

*A. calcoaceticus* M1B (Figure 4) showed 95% biodegradation of butylbenzene (BB) and 53% of *tert*-butylbenzene (TB) after 7 days in control samples which did not contain sand or plant extract. High biodegradation rates might be correlated to biosurfactant production by this strain, enabling effective hydrocarbons adhesion and transport through the membrane. For both hydrocarbons their residual concentration after 14 days of cultivation did not change. The addition of plant extract or sand to the microbial culture contributed to the decline of biodegradation of BB. The highest biodegradation decline was noted in the samples with the addition of *Sapindus mukorossi* extract, both in cultures with and without the addition of solid matrix, in which the biodegradation reached 46% and 48% after 7 days, respectively. Lower biodegradation rates compared to control samples might be correlated with cell membrane sealing and worse hydrocarbon transport through the membrane. Also, the cells’ electrokinetic potential and hydrophobicity did not support effective adhesion of hydrocarbons, which might be attributed to competitive biosurfactant–saponins interactions on the cells’ surface. The observed decrease in biodegradation of BB in the presence of sand and surfactants could be interpreted as being a result of sorption of surfactants to sand particles which, in turn, decreased the bioavailability of saponins for micellar solubilization [35]. Another explanation of decreased biodegradation in the presence of *S. mukorossi* extract would be the fact that the constituents of the plant extract could be toxic for bacterial cells and inhibit their growth. On the other hand, the increase in biodegradation in the presence of *S. officinalis* extract could be justified by the extract stimulation of cells growth and being the additional carbon source for bacterial cells [52].

As it is a hydrocarbon with a branched side chain, *tert*-butylbenzene was not degraded as efficiently as butylbenzene in control samples. In the system with this compound, the addition of sand and saponins was beneficial mostly after 14 days observation. However, the highest increase in biodegradation, up to 77% after 7 days and 89% after 14 days of cultivation, was observed in the sample containing additionally only solid matrix. According to the results presented in Section 2.2., this could be partially attributed to hydrocarbons adsorption on sand particles, thus resulting their removal from the culture medium, but also in a decrease in bioavailability. Analysis of the results of the addition of plant extract revealed the saponins from *S. officinalis* were more effective; in the sample without the addition of sand, biodegradation of TB was 40% after 7 days, however, it increased to 90% after 14 days. In the sample containing both *S. officinalis* extract and sand, the biodegradation was 64% regardless of cultivation time. Observed increase in hydrocarbons removal might be correlated not only to their biodegradation, but also to pollutant emulsification in surfactants micelles, due to presence of both plant- and microbial-derived surfactants in these samples.

In the presence of *Raoultella planticola* M01 (Figure 5), the biodegradation of BB in control samples reached 77% after 7 days and 94% after 14 days. As for the biodegradation of TB by *R. planticola* M01, the results in control sample were similar in the 7-day and 14-day culture and reached about 86%.

For the strain analyzed, the addition of plants extracts did not substantially change the BB and TB biodegradation efficiency. The addition of *Sapindus mukorossi* extract contributed to the increase in biodegradation of BB up to 88% after 7 days, however, the most valuable results were obtained when both *S. mukorossi* extract and sand were present in the microbial culture. In the system in question, biodegradation reached 93% in the 7-day culture. The presence of *S. officinalis*, similarly as sand, did not modify the biodegradation efficiency of BB. On the other hand, the addition of saponins to the microbial culture with TB caused a decrease in biodegradation after 7 days of cultivating and after 14 days, the removal of TB was at the same level as in control sample. It should also be noted that a more permeable cell membrane may improve biotransformation of hydrocarbons due to their better transfer across the membrane [53]. Our results seem to confirm this conclusion, as the permeability of microbial cells was higher in the samples with better degradation efficiency than in those with worse degradation rate.

In general, there is not much literature available regarding the biodegradation of butyl- and *tert*-butylbenzene. Smith and Ratledge [54] have published an article describing the ability of *Pseudomonas* sp. NCIB 10643 to grow on alkylbenzenes as a sole source of carbon and energy. Moreover, it was found that filamentous fungus isolated from Antarctic soil was capable of degrading butylbenzene in 42% [55]. Considering the negative impact of alkane-substituted benzene derivatives on living organisms and their unclear environmental fate, further research in this area is required.

## 3. Conclusions

The present study was designed to determine the impact of butylbenzene and *tert*-butylbenzene adsorption onto sand particles on their biodegradation process, as well as to check the effect of saponins addition to these systems. The kinetics of both hydrocarbons biodegradation was better described by the pseudo-second order model with correlation coefficients over 0.99. In addition, the Freundlich isotherm model was shown to be the most appropriate to characterize the adsorption of butylbenzene and *tert*-butylbenzene on sand.

The investigation of bacteria cell surface parameters has shown differences between surfactant-cultured and unaltered cells, while the analyzed properties of modified cells changed in the direction of improved affinity to hydrocarbons biotransformation. Finally, the beneficial influence of the presence of sand and/or *S. officinalis*saponins on hydrocarbons degradation was evidenced. Only in the case of biodegradation of butylbenzene with *R. planticola* M01, the addition of sand and/or surfactant did not noticeably influence the high initial degradation rate.

The results obtained indicate that the hydrocarbons’ affinity to solid particles should not be neglected in bioremediation processes, and sorption on sand particles plays a significant role in their degradation. Furthermore, the addition of saponins-containing plant extracts alters the bacteria cell surface properties prompting higher degradation rates which might be also affected by the presence of sand particles and diminished hydrocarbon bioavailability. In view of the above, further experiments on the microbial degradation of benzene derivatives in three-phase systems are required.

## 4. Materials and Methods

### 4.1. Strains, Surfactants and Chemicals

Hydrocarbons-degrading bacteria *Raoultella planticola* M01 and *Acinetobacter calcoaceticus* M1B, isolated from a long-term hydrocarbon-contaminated soil by the method described by [30], were used in the experiments. The bacteria were identified genetically, and their nucleotide sequences are available in GenBank under the accession numbers: KX.667738.1 (M01) and KU.563543.1 (M1B).

Mineral salt medium (MSM) containing 7.03 g Na_2_HPO_4_∙12H_2_O, 1.00 g KH_2_PO_4_, 1.00 g (NH_4_)_2_SO_4_, 0.17 g MgSO_4_, 0.008 g Ca(NO_3_)_2_∙4H_2_O, 0.01 g (NH_4_)_5_Fe(C_6_H_4_O_7_)_2_ was used for bacteria cultivation. Each microbial culture was supplemented with the trace elements solution which consisted of 0.3 g L^−1^ H_3_BO_3_, 0.1 g L^−1^ ZnSO_4_∙7H_2_0, 0.03 g L^−1^ Na_2_MoO_4_, 0.03 g L^−1^ MnCl_2_∙4H_2_O, 0.02 g L^−1^ NiCl_2_∙6H_2_O, 0.01 g L^−1^ CuCl_2_∙2H_2_O, 0.2 g L^−1^ CaCl_2_∙6H_2_O. The above-mentioned salts were purchased from Avantor (Gliwice, Poland). Glucose (G) was purchased from BTL (Łódź, Poland) and used as 20% solution in MiliQ water. Analytical grade butylbenzene (BB), *tert*-butylbenzene (TB), hexane and acetone were purchased from Sigma-Aldrich (Poznań, Poland).

The surface active plant extracts of *Sapindus mukorossi* and *Saponaria officinalis*, were obtained as described previously [30,56]. Portions of 1.88 g of *S. mukorossi* and 4.00 g of *S. officianalis* freeze-dried extracts were dissolved in 100 mL of MiliQ water, filtered (0.2 μm) and pasteurized (65 °C, 20 min). The solutions prepared in this way were used in further experiments.

Sand samples were obtained from Baltic seaside beach in Kołobrzeg (54°11′23′’ N, 15°36′11′’ E), washed three times with MiliQ water and acetone. Next, the samples were oven dried (Wamed, Warszawa, Poland) at 200 °C for 24 h, sieved through a metal sieve and the fraction containing particles of sizes 500 to 250 μm was selected.

### 4.2. Microbes Surface Properties Measurement: Cell Surface Hydrophobicity, Zeta Potential and Membrane Permeability

*A. calcoaceticus* M1B and *R. planticola* M01 were cultured in MSM at 30 °C on a rotary shaker at 120 rpm for 6 days. The cultures were consisted of: 18 mL of MSM medium; 2 mL of bacterial inocula (3-days glucose cultures with OD_600_~1.0) and carbon source: plant extract (the quantity corresponding to the final concentration of 1CMC in the culture) or 50 ppm of appropriate hydrocarbon, whereas the control samples were supplemented with 200 μL of 20% glucose solution. Subsequently, the cells were centrifuged at 5000 rpm for 10 min and washed three times with sterile MSM medium before use. Cells were re-suspended in sterile MSM medium to optical density OD_600_~1.0 and used for measurements of microbes’ properties under aerobic conditions. Cell surface hydrophobicity was measured using cell adhesion to hexadecane (MATH method), as described earlier by [56]. For zeta potential evaluation, a 5 mL cell suspension was measured using the ZetaSizerNano ZS (Malvern Instruments Ltd., Malvern, UK) and the electrokinetic potential was calculated from the Smoluchowski equation on the basis of the electrophoretic mobility measurement [57,58]. Membrane permeability was determined by measuring the concentration of *β*-galactosidase released into the solution using *o*-nitrophenyl-*β*-d-galactoside (ONPG) as a substrate [59].

### 4.3. Adsorption Kinetics Modeling

To determine the adsorption–desorption equilibrium, sand samples (3.0 g) were spiked with 10 mL of 1.18 g L^−1^ of butylbenzene or 2.95 g L^−1^ of *tert*-butylbenzene solution upon agitation. Samples (1 mL) were taken after 3, 5, 10, 20, 40, 60, 80, 100 and 120 min and extracted with hexane (1 mL). The amount of hydrocarbon that was adsorbed onto sand particles ($C_{S}^{ads}$, mg g^−1^) was evaluated indirectly, by determining the difference between the initial and the remaining concentration of butylbenzene or *tert*-butylbenzene in aqueous solution ($C_{aq}^{ads}$, mg L^−1^) (Equation (1)).

(1) $$C_{S}^{ads}=\frac{V_{0}}{m_{S}}\times \left(C_{0}-C_{aq}^{ads}\right)$$

where $V_{0}$ is the initial volume of solution (mL); $m_{S}$ is the mass of the sand (g); $C_{0}$ is the initial concentration of hydrocarbon in solution (mg L^−1^). The equilibrium concentration time was defined on the basis of time changes in hydrocarbons concentration in the samples.

For the construction of sorption isotherms, the selected hydrocarbons were added to 3.0 g of sand samples in such a quantity to obtain the final concentrations corresponding to 0, 3, 5, 7, 10, 15, 17 and 20 multiplications of each hydrocarbon water solubility (11.8 mg L^−1^ and 29.5 mg L^−1^ for butylbenzene and *tert*-butylbenzene, respectively). All experiments were carried out in glass test tubes. The mixtures were agitated in a shaker for appropriate time to reach apparent adsorption–desorption equilibrium. All analyses were performed in duplicate for each concentration point.

### 4.4. Hydrocarbons Biodegradation

Bacterial inoculums were prepared in 100 mL of MSM with 1 mL of 20% glucose solution for 72 h on a rotary shaker 120 rpm at 28 °C. Biomass was harvested by centrifugation and washed three times with sterile MSM. Biodegradation tests were carried out in six different sets of experiments, separately for 7 and 14 days: with hydrocarbons only (C); with *S. mukorossi* extract (Sm); with *S. officinalis* extract (So); with sand only(S); with sand and *S. mukorossi* extract (S+Sm); with sand and *S. officinalis* extract (S+So). The experiments were performed in 100 mL Duran^®^ glass bottles. Each bottle contained 18 mL of MSM medium, 2 mL of bacterial inocula (cells re-suspended in MSM after centrifugation and washing), selected hydrocarbon in final concentration of 50 ppm and, if applicable, plant extract solution(110 μL of *S. mukorossi* extract or 500 μL of *S. officinalis* extract); total of 24 vessels. The hydrocarbons in samples after a predetermined time were extracted with 5 mL of hexane and 1 mL samples were analyzed using GC-MS/MS chromatograph (Pegasus 4D, GCxGC-TOMFMS, LECO, St Joseph, MI, USA) with a BPX-5 column (28 m, 250 μm, 0.25 μm).The analyses were performed with helium as a carrier gas (1 mL min^−1^) for 10 min under isothermal conditions (130 °C for butylbenzene and 120 °C for *tert*-butylbenzene).The quantity of hydrocarbons in the samples was determined on the basis of the calibration curve made using measurements for analogous abiotic samples (medium and hydrocarbons only, in concentration of 10, 20, 30, 40, 50, 60 ppm) with known hydrocarbon contents.

### 4.5. Statistical Analysis

All experiments were performed in triplicate (unless otherwise indicated) and the mean values were used in the calculations. Statistical analysis of the correlation of the results were performed using one-way analysis of variance (ANOVA), SigmaPlot 11.0 with the *p* < 0.001.

## Figures and Tables

**Table 1:** Kinetic and sorption isotherm models used for calculations.

Kinetic Model	Equation	Isotherm Model	Equation
Pseudo-first order	$q_{t}=q_{e}\left[1-exp^{-k_{f}t}\right]$	Langmuir	$\frac{C_{s}}{q_{e}}=\frac{1}{q_{m}K_{L}}+\frac{C_{S}}{q_{m}}$
Pseudo-second order	$q_{t}=\frac{k_{s}q_{e}^{2}t}{1+k_{s}q_{e}t}$	Freundlich	Cs=KF×Caq1n
			logCS=logKF×1nlogCaq
Intraparticle diffusion	$q_{t}=k_{id}\sqrt{t}+I$		

where: *t*—is contact time (min); *q_t_*—is the amount of adsorbed hydrocarbon adsorbed at time (mg g^−1^); *k_f_*—is the pseudo-first order rate constant (min^−1^); *k_S_*—is the pseudo-second order rate constant(g mg^−1^ min^−1^); *k_id_*—is the intraparticle diffusion rate constant (mg g^−1^ min^−1/2^); *C_S_*—is the concentration of hydrocarbon sorbed on the soil at the sorption equilibrium time (mg g^−1^); *C_aq_*—is the concentration of hydrocarbon in the aqueous phase at sorption equilibrium time (mg L^−1^); *K_F_*—is the Freundlich sorption coefficient (mg g^−1^) and 1/*n* is the Freundlich exponent, a constant describing the strength of sorption; *q_e_*—adsorption capacity (mL g^−1^); *q_m_*—maximum sorption capacity (mg g^−1^); *K_L_*—Langmuir equilibrium constant (mL mg^−1^).

**Table 2:** Kinetic parameters calculated for butylbenzene and *tert*-butylbenzene sorption process on sand particles.

Kinetic Model	Hydrocarbon	Parameters	Value	*R* ^2^
Pseudo-first order	BB	*k_f_* (min^−1^)	0.04606	0.924
		*q_e_* (mg g^−1^)	15.7398	
	TB	*k_f_* (min^−1^)	0.03685	0.972
		*q_e_* (mg g^−1^)	25.8821	
Pseudo-second order	BB	*k_s_* (g mg^−1^ min^−1^)	0.09721	0.992
		*q_e_* (mg g^−1^)	1.84502	
		*h*_0_ (mg g^−1^ min^−1^)	0.33091	
	TB	*k_s_* (g mg^−1^ min^−1^)	0.03009	0.998
		*q_e_* (mg g^−1^)	6.53595	
		*h*_0_ (mg g^−1^ min^−1^)	1.28535	
Intraparticle diffusion	BB	*k_id_* (mg g^−1^ min^−0.5^)	0.030	0.460
	TB	*k_id_* (mg g^−1^ min^−0.5^)	0.251	0.741

**Table 3:** Kinetic parameters of adsorption of selected hydrocarbons onto sand particles calculated assuming different models of isotherms.

Isotherm Model	Hydrocarbon	Parameters	Value	*R* ^2^
Langmuir	BB	*a* (mL mg^−1^)	2.15054	0.916
		*b* (mL g^−1^)	2.59777	
	TB	*a* (mL mg^−1^)	0.59595	0.982
		*b* (mL g^−1^)	5.21118	
Freundlich	BB	*K_F_* (mg g^−1^)	0.478	0.987
		*n*	1.20048	
	TB	*K_F_* (mg g^−1^)	0.074	0.997
		*n*	1.27064	
